# The Additional Error of Inertial Sensors Induced by Hypersonic Flight Conditions

**DOI:** 10.3390/s16030299

**Published:** 2016-02-26

**Authors:** Volodimir Karachun, Viktorij Mel’nick, Igor Korobiichuk, Michał Nowicki, Roman Szewczyk, Svitlana Kobzar

**Affiliations:** 1 Department of Biotechnology and Engineering, National Technical University of Ukraine “Kyiv Polytechnic Institute”, 37, Avenue Peremogy, Kyiv 03056, Ukraine; karachun11@i.ua (V.K.); bti@fbt.ntu-kpi.kiev.ua (V.M.); 2 Industrial Research Institute for Automation and Measurements PIAP, Jerozolimskie 202, 02-486 Warsaw, Poland; kiv_Igor@list.ru; 3 Institute of Metrology and Biomedical Engineering, Warsaw University of Technology, Boboli 8, 02-525 Warsaw, Poland; r.szewczyk@mchtr.pw.edu.pl; 4 Foreign Languages Department, Zhytomyr State Technological University, Chernyakhovskogo str., 103, Zhуtomуr 10005, Ukraine; sk-8@ukr.net

**Keywords:** hypersonic technologies, inertial sensors, additional acoustic error

## Abstract

The emergence of hypersonic technology pose a new challenge for inertial navigation sensors, widely used in aerospace industry. The main problems are: extremely high temperatures, vibration of the fuselage, penetrating acoustic radiation and shock N-waves. The nature of the additional errors of the gyroscopic inertial sensor with hydrostatic suspension components under operating conditions generated by forced precession of the movable part of the suspension due to diffraction phenomena in acoustic fields is explained. The cause of the disturbing moments in the form of the Coriolis inertia forces during the transition of the suspension surface into the category of impedance is revealed. The boundaries of occurrence of the features on the resonance wave match are described. The values of the “false” angular velocity as a result of the elastic-stress state of suspension in the acoustic fields are determined.

## 1. Introduction

This paper is related to the field of applied mechanics and devoted to the study of polyaggregate inertial sensors under the operating conditions of hypersonic motion [1]. Gyroscope suspension is a polyaggregate structure, *i.e.*, an insulated cylindrical body containing a heavy liquid; the heavy liquid contains a sealed float made of a light metal (e.g., magnesium alloy), and inside the float there is a gyro unit surrounded by an inert gas. The influence of the penetrating acoustic radiation on the additional error of the two-stage floating gyroscope is studied [2]. Establishing the nature of this phenomenon will allow the selection of effective methods to suppress its influence and will ensure the required accuracy of air navigation [3,4].

On 9 January 2015 the “Club of the Hypersonic Weapon Owners” added another participant. The Peoples Republic of China tested a hypersonic glider called WU-14. It is a controlled device, which is mounted on the aerodynamic payload fairing of a ballistic missile. The missile lifts the glider into space, and then it dives on the target, developing a speed of thousands of kilometers per hour.

According to the Pentagon, the Chinese hypersonic machine WU-14 can be mounted on a variety of Chinese ballistic missiles with a shooting range from 2 to 12,000 km. During the January tests, it developed a speed of 10 Mach. Modern air defense systems are not able to reliably hit a target maneuvering with such speed. Thus, China became the third nation, after the United States and Russia, to possess hypersonic technology.

The benefits of such technology are due to the long shooting range (up to a global attack on any part of the Earth’s surface), a fairly simple device unit (no boosters are needed), a large warhead mass and finally, high speed of the flight—more than 10 Mach.

The Russian Federation is focused on developing missiles with hypersonic ramjet engines. There is a Russian-Indian project to develop such weapon systems. Thus, by 2023 India will also be able to enter the “Hypersonic Club.” The advantage of hypersonic missiles lies not only in a lower cost, but also greater flexibility in application technology than gliders launched by ballistic missiles.

Both types of hypersonic weapons can carry a conventional or nuclear warhead. Experts of the Australian Strategic Policy Institute calculated that the kinetic energy of the impact of a hypersonic warhead without a high-explosive or nuclear warhead, with mass of 500 kg and a speed of 6 Mach is comparable to the devastation caused by the the explosion of the warhead of an ordinary *AGM-84 HARPOON* subsonic missile, equipped with a warhead with explosives weighing about 100 kg.

It may seem that the hypersonic weapons are not much superior to the existing supersonic ones, however, it is not so simple. The fact is that the ballistic missile warheads are easily located at a considerable distance and, moreover, travel along a predictable trajectory. While their speed is huge, modern computer technologies have made it possible to intercept warheads during their descent along a ballistic curve as the US missile defense system successfully demonstrated.

On the other hand, hypersonic aircraft come at a target along a relatively flat trajectory, they are in the air for very little time and, what is more important, have high maneuverability. Modern air defense systems are not able to detect, classify, and then hit the hypersonic target in such a short period of time. For example, the *S-300* anti-aircraft missile system can accelerate to a speed of 7.5 Mach, and even then, only for a short period of time. Thus, it becomes apparent that any target flying at a speed of about 10 Mach, in most cases, would be “too tough”.

The benefits provided by hypersonic technologies, as it turns out, also generate a lot of air navigation problems such as: extremely high temperatures—up to 2000 °C, the fuselage vibration, control problems, penetrating acoustic radiation and shock N-waves. This paper focuses on the study of the elastic effects of the last two factors on the aircraft, in particular, on their impact on the on-board navigation equipment.

Inertial navigation devices are widely used in many industries, particularly in rocket and space technology [5,6,7,8,9,10]. This demand can be explained by the remarkable property of inertial systems, peculiar only to them–autonomy [11,12].

Sometimes in the literature critical remarks appear about the capabilities of inertial navigation systems for modern technologies [13]. Various “exotic” sensor schemes for positioning tasks, including solid-state sensors are offered, but the latter are totally unsuitable for sub-orbital and atmospheric hypersonic technologies due to the extremely high temperatures and powerful shock N-waves of full-scale real life conditions.

The aim of this work was to study the effect of spatial (geometric) resonance in a hypersonic aircraft motion conditions based on the emergence of additional autonomous positioning errors being one of the causes of the technological risks of operational use [14]. The most complicated inertial sensor suspension—the polyaggregate one—is under consideration.

## 2. Structural Diagram of the Floating Gyroscope

**Figure 1:**
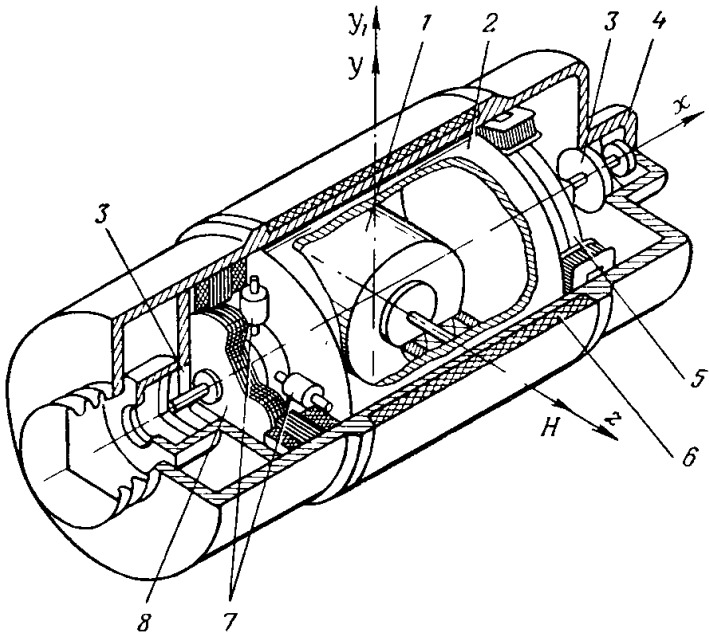
Structural diagram of the floating gyroscope: 1-gyromotor; 2-float; 3-bearings; 4-body; 5-torque sensor; 6-thermostat winding; 7-calibration nuts; 8-angle sensor.

A floating gyroscope is a gyroscope with two degrees of freedom, where torque proportional to rotation rate of gyroscope resists its rotation about the gimbal axis [3]. The basic structural diagram of the floating integrating gyroscope is shown in simplified form in Figure 1.

Gyromotor 1 of the device is rigidly fixed in an airtight cylindrical float 2 and forms a gyro unit. The floating gyro unit of the device is mounted on bearings 3 placed in a pressurized body 4 of the device so that it can rotate relative to the body of the device around the output axis *x_1_*. The body 4 is cylindrical at a section covering the floating gyro unit. There is a small gap of constant value (0.1–0.2 mm) in the section between the surface of the device body and the cylindrical surface of the float.

The internal space of the body is filled with a high density fluorocarbon fluid (*ρ* = 1.93 ÷ 1.98 g/cm^3^ range) of high viscosity ratio. The fluid is used for hydrostatic unloading of floating gyro unit supports, protection of device supports against shock and vibration and creation of damping torque. Functions of a damper are performed by the outer surface of the float *2*, the inner surface of the body 4 and the fluid in the gap between these surfaces. Fluid viscosity and gap size are selected so that the flow of fluid in the gap remains laminar at almost all possible rates of floating gyro unit turn relative to the device body and that damping torque is strictly proportional to the rate of floating gyro unit turn. Automatic temperature control is provided in the device for maintaining density and viscosity of the fluid.

The winding 6 of the thermostat is wound around the cylindrical surface of the device body. The volume of the floating gyro unit is selected so that its buoyancy in the fluid at the operating temperature is close to zero. Thus, there is almost complete unloading of the supports, which makes it possible to replace ball bearings with jewel bearings and achieve negligibly small support friction torque.

To avoid uncertainty torques from translational acceleration along the output axis the floating gyro unit should be carefully balanced. It is necessary to align the center of gravity of the floating gyro unit with the pressure center of the *x_1_*-axis fluid displaced by it. A gyro unit is balanced with respect to the *x_1_*-axis by four nuts 7, screwed on four mutually perpendicular threaded rods fixed to the axis of the floating gyro unit. Angle sensor 8 is for detection of the floating gyro unit turning angle.

A torque sensor 5 of the floating gyroscope is designed to compensate constant uncertainty torques along output axis of the device, and to apply a control torque about an output axis of the gyroscope at attitude stabilization mode.

**Figure 2:**
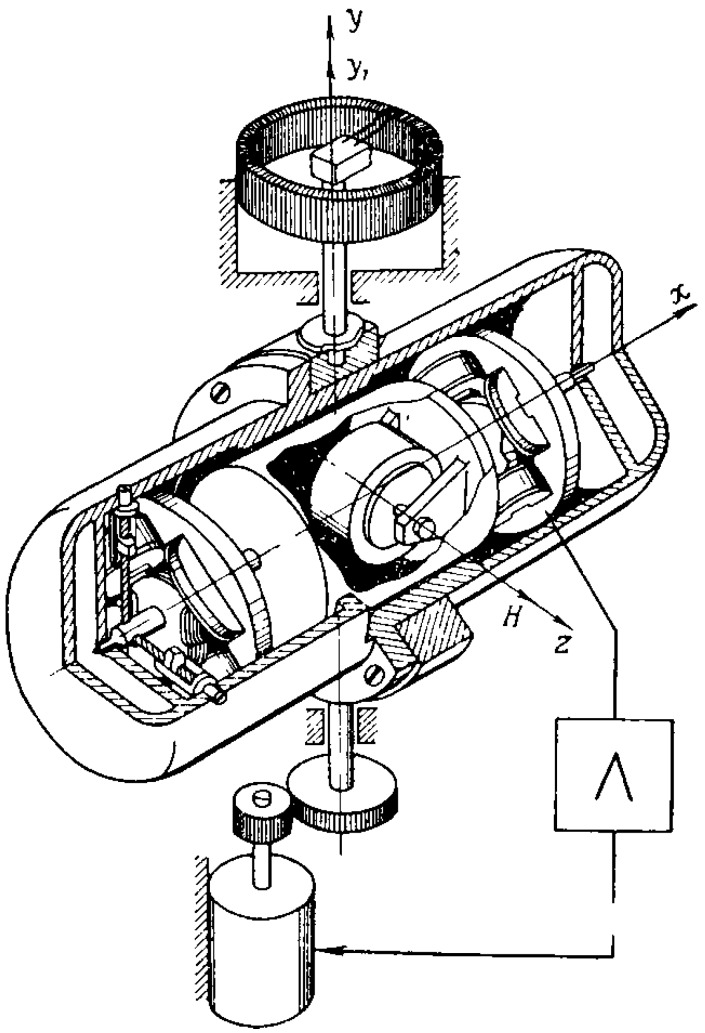
Single-axis gyrostabilizer with a floating gyroscope.

A sensitive axis or a measurement axis is the axis *y* perpendicular to the plane of the gyroscope angular momentum axis and the output axis *x*. The floating gyroscope is mounted on the platform to stabilize it so that its sensitive axis *y* coincides with the stabilization axis *y_1_* (Figure 2).

If the platform with a gyroscope rotates in space about an axis *y_1_* under the action of any external moments, gyroscopic torque ${\bar{M}}_{g}=\bar{H}x{\bar{\omega }}_{y}$ (*H*: gyroscope angular momentum) would affect the floating gyro unit about the axis *x*. Under the action of a gyroscopic torque the floating gyro unit begins to rotate around the axis *x* at an angular velocity $\dot{\beta }$, trying to align the vector *H* with the vector $\omega_{y1}$. At the same time a damping torque would prevent the rotation of the floating gyro unit:

$$M_{d}=B\dot{\beta }$$

where *B* is the float damping coefficient, in mN·cm·s:

$$B=\frac{2\pi R^{3}l_{f}\mu }{98l\delta }$$

where: R-float radius; l_f_-float length; µ-dynamic viscosity coefficient; δ-distance between the shell and the float.

For small angles *β* and constant speed $\omega_{y1}$:

(1) $$B\beta =H\omega_{y1}$$

Integrating Equation (1), we obtain:

(2) $$\beta -\beta_{0}=\frac{H}{B}\int_{o}^{t}\omega_{y}dt$$

The signal from the angle sensor of the floating integrating gyroscope proportional to the angle *β* comes as a control signal for stabilization platform.

The ratio of *H/B* gyroscope angular momentum *H* to damping ratio *B* is referred to as sensitivity of the device or integration constant. In terms of accuracy of platform stabilization, the device integration constant *h = H/B* shall be increased. However, the increase of *h* is limited by strength of floating device jewel bearings.

Let’s consider the precession equations of floating gyroscope motion and equations of platform motion about the stabilization axis.

The equation of gyroscope motion around the axes *x* and *y* has the following form respectively:

(3) $$В\dot{\beta }-H\dot{\alpha }+M_{con}=0$$

(4) $$F_{op}l_{op}-H\dot{\beta }=0$$

and the equation of motion about the stabilization axis:

(5) $$-F_{op}l_{op}-W(s)\beta -B_{1}\dot{\alpha }=0$$

where *M_con_* is control torque along the gyroscope output axis *x*; $\dot{\alpha }$ is angular velocity of the platform along the stabilization axis; *F_op_* is force in floating device supports; *l_op_* is the distance between floating device supports; *W*(*s*) is transfer function of unloading circuit; *B*_1_ is damping factor of stabilization axis.

From Equation (4) we have:

(6) $$F_{op}=\frac{H}{l_{op}}\dot{\beta }$$

Let’s use the value $\dot{\beta }$ from Equation (3) in the Equation (6):

(7) $$F_{op}=\frac{H}{l_{op}}\frac{H\dot{\alpha }-M_{con}}{B}=h\frac{H\dot{\alpha }-M_{con}}{l_{op}}$$

It follows from the Equation (7) that force in the jewel bearings increases with increasing device integration constant *h*. Therefore, integration constant *h* is in 3 ÷ 8 range for real devices.

## 3. The Mathematical Model of Low Frequency Resonance

Let’s analyze the effect of acoustic radiation on series-produced two-stage gyroscope of unified angular velocity sensor (UAVS) class with floating suspension. We consider a circular cylindrical housing of the device as a thin shell of infinite length, assuming that the impact of the ends on the shell part is minimal.

The insulation of the shell from airborne noise is characterized by fraction of incident sound power transmitted through the shell. A sound transmission coefficient *A* is the ratio of the pressure amplitude in the transmitted wave to the pressure amplitude in the incident wave. Since powers transferred by waves are proportional to the squares of pressure amplitudes [15,16,17], the value |А|^2^ is the shell sound transmission coefficient $\tau_{\vartheta }$ at sound wave incidence at angle ϑ.

However, the main interest is the value of sound transmission at the diffusive sound incidence, rather than at the certain value of incidence angle. This is due to the fact that, in practice, one usually has to deal with the sound field close to the diffusive one. shell sound transmission coefficient τ at the diffusive wave incidence, which is statistically the average value of sound transmission at all possible angles of sound incidence, is associated with shell sound transmission coefficient at the wave incidence at angle ϑ. If the shell is only affected by normally applied influence, then the value of the acoustic permeability of the housing will be determined by the relation [15,16,17]:

(8) $$\tau =\frac{1}{{\left|1+\frac{i}{\Delta_{p}}\left[1-{\left(\frac{\omega }{\omega_{c}}\right)}^{2}\sin^{4}\vartheta -{\left(\frac{\omega_{pp}}{\omega }\right)}^{2}\sin^{4}\psi \right]\right|}^{2}}$$

where $\Delta_{p}=\frac{2\rho_{0}c_{0}}{m_{p}\omega \text{cos}\vartheta }$, *c*_0_ is speed of sound in air; *ρ*_0_ is air density (mass per unit volume); *m_p_* is the mass of the plate unit area; *w_pp_* is the angular self-oscillation frequency, purely radial oscillations of the ring of the device housing in compression-tension; *w* is wave circular frequency; $i=\sqrt{-1}$; ϑ is soundwave incident angle; ψ is angle between the plane of incidence and the plane of cylindrical shell cross section; $\omega_{с}=2\pi f_{c}$ is circular cut-off frequency.

Equation (8) shows that the acoustic permeability of the housing can decline sharply at frequencies below the cutoff $f_{c}$.

Then, when the condition:

(9) $$\omega =\omega_{pp}\sin^{2}\psi$$

The shell part of the housing will be *“acoustically transparent”* (of course, assuming no losses). This condition can be considered as equality of the frame circumference trace and the circle wave trace on a plane parallel to the front of the incident wave, *i.e.*,

(10) $$\frac{2\pi R}{\sin\psi }=\lambda_{p}\sin\psi$$

where *R* is soundproofing isolation in *m*; $\lambda_{p}$ is sound wavelength in air.

Thus, in the cylindrical housing of the device, the geometric (spatial) resonance of *circumferential waves* occurs at low frequencies. But only at frequencies not higher than *w_pp_*.

In the case of diffuse radiation by a sound wave, theacoustic permeability coefficient of the housing is determined by averaging the Equation (8) according to Paris, *i.e.*,

(11) $$\underset{}{\tau_{\theta \psi }=}=\int_{0}^{\frac{\pi }{2}}\int_{0}^{\frac{\pi }{2}}\tau \sin2\theta \;\partial \theta \;\partial \psi$$

Thus, it becomes apparent, that the bending vibrations of the housing (Z_a_ << Z_c_), can trigger a resonance as an equality of incident wave and flexural wave traces only at frequencies ω, exceeding the cutoff frequency $\omega_{c}$. Moreover, each frequency ω is corresponded by the match angle $\theta_{c}$.

On the other hand, the circumferential housing vibrations (Z_a_ >> Z_c_) at low frequencies cause equality of the frame circumference trace and the circumferential wave trace on a plane that is parallel to the wave front. Assume the radius *R* of the floating device housing is eequal to 0.025 m, and the speed of the circumferential wave in aluminum equal to c_1_ = 6400 ms^−1^. Then it is possible to set the conditions of low-frequency resonance of the circumferential wave in the gyroscope housing with hydrostatic suspension.

First of all, define the cut-off frequency:

$$f_{c}=\frac{c_{1}}{2\pi R}=\frac{6400}{2\cdot 3.14\cdot 0.025}=40.764\text{ kHz}$$

The values of the angle ψ at which the resonance occurs at low frequencies are determined by the Equation (10).

With decreasing of the angle ψ of wave incidence, the match frequency *f* is also reduced, *i.e.*,

(12) $$2\pi R\frac{f}{c_{1}}=\sin^{2}\psi$$

**Figure 3:**
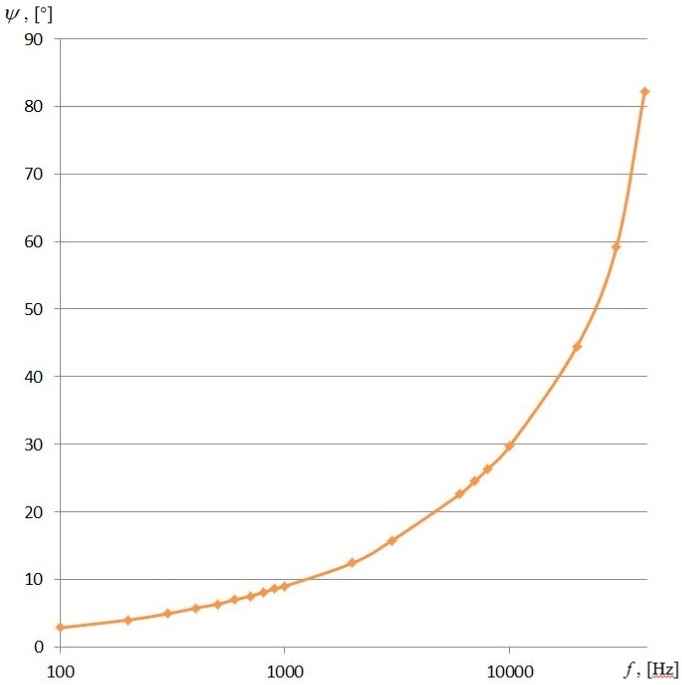
The resonance values of the angle ψ to the circumferential wave. Material–aluminum, speed c_1_ = 6400 ms^−1^.

The resonant values of the angle ψ are e.g., 9° when *f* = 1000 Hz, 12°25’ when *f =* 2000 Hz, 82°10’ when *f =* 40,000 Hz (Figure 3)*.* In the latter case, the device error may exceed 2° s^−1^.

## 4. Diffraction of Sound Waves on the Suspension of the Gyroscope and the Measurement Errors

In case of the geometric resonance the acoustic radiation freely enters the device and hits the surface of the float through hydrostatic suspension. Diffraction of sound waves causes cyclically deformed condition of the surface, the coordinate functions of which will have the form [2]:

(13) $$\begin{array}{l} U_{z}=\sum_{k=0}^{\infty }\left[a_{k}^{\left(1\right)}\left(t\right)z^{2}{\left(1-z\right)}^{2}\text{cos}k\varphi \text{cos}z+a_{k}^{\left(2\right)}\left(t\right)z^{2}{\left(1-z\right)}^{2}\sin k\varphi \sin z\right]\\ U_{\varphi }=\sum_{k=0}^{\infty }\left[b_{k}^{\left(1\right)}\left(t\right)z^{2}{\left(1-z\right)}^{2}\sin k\varphi \text{cos}z+b_{k}^{\left(2\right)}\left(t\right)z^{2}{\left(1-z\right)}^{2}\text{cos}k\varphi \sin z\right]\\ U_{r}=\sum_{k=0}^{\infty }\left[c_{k}^{\left(1\right)}\left(t\right)z^{4}{\left(1-z\right)}^{4}\text{cos}k\varphi \text{cos}z+c_{k}^{\left(2\right)}\left(t\right)z^{4}{\left(1-z\right)}^{4}\sin k\varphi \sin z\right] \end{array}$$

where $a_{k},\;b_{k},\;c_{k}$ are the coefficients, z, φ are the dimensionless coordinates of the float along the meridian line and along the parallel line, respectively.

**Figure 4:**
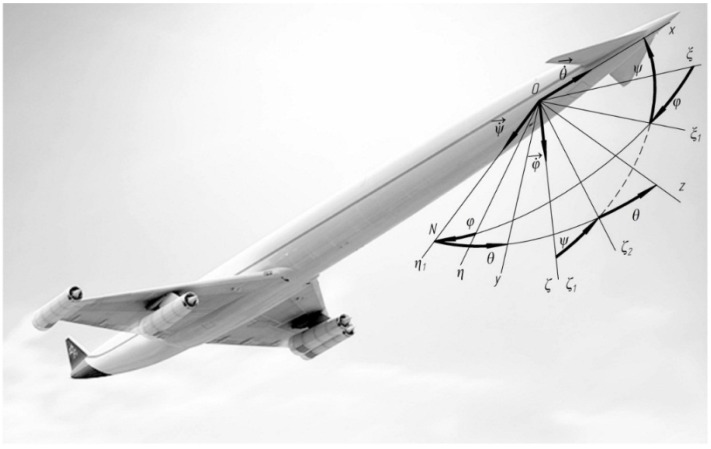
The scheme of the Euler angles (φ: yaw anqle; ψ : pitch anqle; θ : roll anqle).

**Figure 5:**
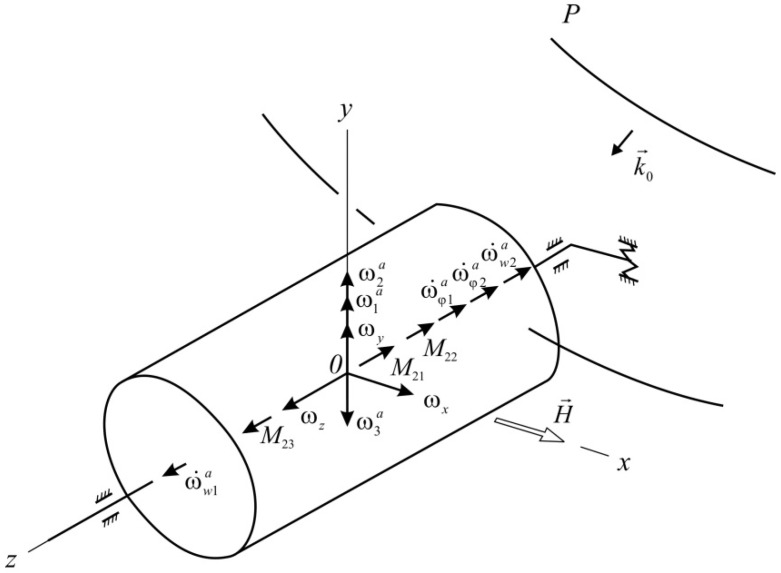
Disturbed state of the gyroscope suspension under sound exposure.

Bind an aircraft with the coordinate system *Ox*_1_*y*_1_*z*_1_ (Figure 4). Bind the float with the non-inertial coordinate system *Oxyz* (Figure 5). Let’s analyse the inciting motion of the float in flight. We will hard bind the system of co-ordinates *Oxyz* with the body of aircraft: *Ox* will be set along the axis of the aircraft, *Oy* and *Oz* will be placed in the former plane. For the supporting system of coordinates we choose the axes which are related to Earth. Axis Оζ will be set vertically downward, axis Оξ-horizontally (for example, directed on the line of the set course), axis Оη constitutes the right three of axes Оξηζ with the first two axes.

Suppose, the aircraft at the start moment occupies an arbitrary position. Through its center of mass of a plane perpendicular to the longitudinal axis (the plane of the frame) to the intersection with the horizontal plane. For the crossing lines of these planes *ON* (lines of knots) we will direct axis $O\eta_{1}$ and will draw in the horizontal plane axis $О\xi_{1}$, perpendicular to $O\eta_{1}$. For the Euler angles we will choose the angle of rotation around the vertical plane of the horizontal coordinate Оξη to match it with the axes of the system $O\xi_{1}\eta_{1}$ (called the yaw angle φ), the angle of rotation around the line of nodes $\xi_{1}O\zeta$ ON of the coordinate plane to align the axis $O\xi_{1}$ with the longitudinal axis of the airplane (called the pitch angle ψ). In this case axis Oζwill take the position of $O\zeta_{1}$ in the frame plane and the angle of rotation of the plane $\eta_{1}O\zeta_{1}$ about the longitudinal axis of the fuselage Ox (angle of roll θ). The corresponding angular velocities will be directed along the vertical line $\left(\vec{\dot{\varphi }}\right)$, the line of nodes $\left(\vec{\dot{\psi }}\right)$ and along the axis of the vehicle $\left(\vec{\dot{\theta }}\right)$.

The angular velocity of the aircraft can be expressed as an expansion in the unit vectors ${\vec{e}}_{1},{\vec{e}}_{2},{\vec{e}}_{3}$ of the axes $O\eta_{1}$, Oζ and Ox:

$$\vec{\omega }={\vec{e}}_{1}\dot{\varphi }+{\vec{e}}_{2}\dot{\psi }+{\vec{e}}_{3}\dot{\theta }$$

or in projections on the axis associated with the aircraft body:

$$\vec{\omega }={\vec{e}}^{x}\omega_{x}+{\vec{e}}^{y}\omega_{y}+{\vec{e}}^{z}\omega_{z}$$

Specify the position of the axes associated with the aircraft by Euler angles (Figure 4). As the reference coordinate system Oξηζ select three axes, connected, for example, with the Earth. Then, the projections of the angular velocity on the axis *Ox*_1_*y*_1_*z*_1_ and on the axis *Oxyz* will be determined by the ratio:

(14) $$\begin{array}{l} \omega_{x_{1}}=\dot{\theta }-\dot{\varphi }\sin\psi \\ \omega_{y_{1}}=\dot{\varphi }\sin\theta \text{cos}\psi +\dot{\psi }\text{cos}\theta \\ \omega_{z_{1}}=\dot{\varphi }\text{cos}\theta \text{cos}\psi -\dot{\psi }\sin\theta \end{array}$$

(15) $$\begin{array}{l} \omega_{x}=\omega_{x_{1}}\text{cos}\beta +\omega_{y_{1}}\sin\beta \\ \omega_{y}=-\omega_{x_{1}}\sin\beta +\omega_{y_{1}}\text{cos}\beta \\ \omega_{z}=\omega_{z_{1}}+\dot{\beta } \end{array}$$

and θ=θ(t), ψ=ψ(t), φ=φ(t).

In the conditions of a three-axis swinging of AC, the impedance surface of the float generates moments of the Coriolis inertia forces [18], which the gyroscope responds to. Moreover, the moments when the vectors are directed along the input axis of the gyroscope directly cause the precession of the movable part of the device with an angular velocity ${\left(\omega_{\varphi }^{a}\right)}_{1}$, ${\left(\omega_{\varphi }^{a}\right)}_{2}$, ${\left(\omega_{r}^{a}\right)}_{1}$, ${\left(\omega_{r}^{a}\right)}_{2}$:

(16) $$\begin{array}{c} {\left(\omega_{\varphi }^{a}\right)}_{1}=\frac{8\omega_{x}I_{z}i\omega_{1}b_{1}^{\left(2\right)}z^{2}{\left(1-z\right)}^{2}\text{exp}i\omega_{1}t\sin z}{3HR}\\ {\left(\omega_{\varphi }^{a}\right)}_{2}=\frac{8\omega_{y}I_{z}i\omega_{1}b_{1}^{\left(1\right)}z^{2}{\left(1-z\right)}^{2}\text{exp}i\omega_{1}t\text{cos}z}{3HR}\\ {\left(\omega_{r}^{a}\right)}_{1}=-\frac{8\omega_{x}I_{z}i\omega_{1}c_{1}^{\left(1\right)}z^{4}{\left(1-z\right)}^{4}\text{exp}i\omega_{1}t\text{cos}z}{3HR}\\ {\left(\omega_{r}^{a}\right)}_{2}=\frac{8\omega_{y}I_{z}i\omega_{1}c_{1}^{\left(1\right)}z^{4}{\left(1-z\right)}^{4}\text{exp}i\omega_{1}t\text{cos}z}{3HR} \end{array}\}$$

where *H, I_z_* are the angular momentum of the gyroscope and the moment of inertia of the float respectively.

The moments of the Coriolis inertia forces with vectors which are directed along the axis of the gyroscope precession (axis *Oz*) will cause a reaction that will generate “false” angular velocities in the input axis *Oy* of the device:

(17) $$\begin{array}{c} \omega_{1}^{a}=\frac{4\pi \omega_{x}I_{z}i\omega_{1}\text{exp}i\omega_{1}ta_{1}^{\left(1\right)}z^{2}{\left(1-z\right)}^{2}\text{cos}z}{HR}\\ \omega_{2}^{a}=\frac{4\pi \omega_{y}I_{z}i\omega_{1}\text{exp}i\omega_{1}ta_{1}^{\left(2\right)}z^{2}{\left(1-z\right)}^{2}\sin z}{HR}\\ \omega_{3}^{a}=\frac{-8\omega_{z}I_{z}i\omega_{1}\text{exp}i\omega_{1}tc_{1}^{\left(2\right)}z^{4}{\left(1-z\right)}^{4}\text{cos}z}{HR} \end{array}\}$$

Thus, the suspension of the gyroscope comprises three cylinders: a housing, a hydrostatic cylinder suspension and a float. Geometric resonance of the housing reduced the energy loss of the acoustic emission to zero and the sound wave without dissipation begins to interact with the liquid, which for its part turns from a static into an active energy substance due to the wave matches for flexural and circumferential waves in the housing, and also forms the “caustic” zone, confocal axis of the device precession. Nonlinear oscillations of the weighing liquid and a sound wave create an elastic-stress state of the float, which is perceived by the gyroscope as an input value, *i.e.*, the measured angular velocity, actually being “false”.

Experimental studies of the floating gyroscope were carried out at the Sirena installation of the Institute for Strength Issues of the National Academy of Sciences of Ukraine and in the Chinese 3560 ultrasonic installation. The results are published in [19,20,21,22].

## 5. Conclusions

Benchmark tests have allowed us to determine the maximum value of the acoustic error. For example, for a serial device UAVS 2-6AS intended to be used in aircraft, the worst-case resonance error exceeded 2° s^−1^. It is a major flaw, for the device sensitivity of 0.09° s^−1^ and limit of effective range of 6° s^−1^. Obviously, there is a question about the functional ability of two-stage gyroscopic angular velocity sensors under the operating conditions of hypersonic motion.

Such flight and navigation instrument errors create an exceptional technological risk, for example, while performing the docking maneuver, a smooth landing on a runway or on the mobile base platform.

The interpretation of the nature of acoustic error appearance leads to the conclusion about the necessity to create on the aircraft “an acoustic comfort zone” for aircraft instrumentation. What methods will be used—active, passive, auto compensational or others—should be settled in every case on the basis of performance or mass and overall requirements. These can equally apply to both controlled and automatic flights. The easiest way to reduce an additional error is to provide noise insulation to a gyroscope (passive sound insulation methods). There are schematic solutions to reduce the effect of sound waves: self-compensation methods, forced gimbal rotation method, double-channel self-compensation method. An effective way to prevent the influence of sound waves is represented by an *active* method, which consists in the formation of the same sound field, but in the opposite phase, which can significantly reduce the perturbing sound field.
